# Overlapping pathogenic de novo CNVs in neurodevelopmental disorders and congenital anomalies impacting constraint genes regulating early development

**DOI:** 10.1007/s00439-022-02482-5

**Published:** 2022-11-16

**Authors:** Seyed Ali Safizadeh Shabestari, Nasna Nassir, Samana Sopariwala, Islam Karimov, Richa Tambi, Binte Zehra, Noor Kosaji, Hosneara Akter, Bakhrom K. Berdiev, Mohammed Uddin

**Affiliations:** 1 Mohammed Bin Rashid University of Medicine and Health Sciences, Dubai, UAE; 2 University of Guelph, Toronto, ON Canada; 3 University of Bremen, Bremen, Germany; 4 Genetics and Genomic Medicine Centre, NeuroGen Healthcare, Dhaka, Bangladesh; 5 GenomeArc Inc, Toronto, ON Canada

## Abstract

Neurodevelopmental disorders (NDDs) and congenital anomalies (CAs) are rare disorders with complex etiology. In this study, we investigated the less understood genomic overlap of copy number variants (CNVs) in two large cohorts of NDD and CA patients to identify de novo CNVs and candidate genes associated with both phenotypes. We analyzed clinical microarray CNV data from 10,620 NDD and 3176 CA cases annotated using Horizon platform of GenomeArc Analytics and applied rigorous downstream analysis to evaluate overlapping genes from NDD and CA CNVs. Out of 13,796 patients, only 195 cases contained 218 validated de novo CNVs. Eighteen percent (31/170) de novo CNVs in NDD cases and 40% (19/48) de novo CNVs in CA cases contained genomic overlaps impacting developmentally constraint genes. Seventy-nine constraint genes (10.1% non-OMIM entries) were found to have significantly enriched genomic overlap within rare de novo pathogenic deletions (*P* value = 0.01, OR = 1.58) and 45 constraint genes (13.3% non-OMIM entries) within rare de novo pathogenic duplications (*P* value = 0.01, OR = 1.97). Analysis of spatiotemporal transcriptome demonstrated both pathogenic deletion and duplication genes to be highly expressed during the prenatal stage in human developmental brain (*P* value = 4.95 X 10^–6^). From the list of overlapping genes, *EHMT1*, an interesting known NDD gene encompassed pathogenic deletion CNVs from both NDD and CA patients, whereas *FAM189A1*, and *FSTL5* are new candidate genes from non-OMIM entries. In summary, we have identified constraint overlapping genes from CNVs (including de novo) in NDD and CA patients that have the potential to play a vital role in common disease etiology.

**Supplementary Information:**

The online version contains supplementary material available at 10.1007/s00439-022-02482-5.

## Introduction

Neurodevelopmental disorders (NDDs) and congenital anomalies (CAs) are commonly reported as a collection of rare disorders with a strong genetic basis (Casanova et al. 2018; Akter et al. 2021). NDDs are characterized by disruptions in tightly coordinated events of brain development that hinder achieving emotional, cognitive, and motor developmental milestones (Parenti et al. 2020). For example, gene mutations that occur in synaptic proteins, neurexin 1 (*NRXN1*) and *SHANK3* have been associated with the development of autism in early childhood (Walsh et al. 2008). NDDs constitute attention deficit hyperactivity disorder (ADHD), intellectual disability (ID), communication disorders, epilepsy, and autism spectrum disorder (ASD) (Mullin et al. 2013; Hu et al. 2014; Nassir et al. 2021). In contrast, CAs include a broad range of visible abnormalities of body structure or function that exist at birth with a prenatal origin (World Health Organization 2020). CAs are a broad umbrella of disorders consisting of congenital heart defects (CHDs), microcephaly, and dysmorphic features such as cleft palate among others (Dolk et al. 2010; Duncan and Chodirker 2011; Kaminsky et al. 2011; DeSilva et al. 2016; Ameen et al. 2018).

Although there is no universally accepted phenotypic criteria to differentiate these two broad pathologies (Sugranyes et al. 2011; Owoeye et al. 2013; Toufaily et al. 2018), they are further categorized into different disease entities based on phenotype since there are no specific biomarkers to diagnose or differentiate between different NDDs and CAs (American Psychiatric Association 2013). However, they are strongly interlinked through their phenotypic pathogenesis and complications. For instance, patients with CHDs are at higher risk of developing NDDs, with a 20% chance of progression for mild CHD patients, and a higher than 50% probability for severe cases (Marino et al. 2012). This is likely a result of poor defected blood flow to the brain that compromises oxygen delivery, in turn affecting brain development (Perles et al. 2015; Ta-Shma et al. 2018).

To better understand the overlap of such phenotypes, it is important to investigate the underlying genomic interrelations. There is a host of genomic disorders (Bragin et al. 2014; Uddin et al. 2016) related to large structural variants that often present phenotypes that manifest with different disorders. For example, 15q13.3 microdeletion syndrome manifests in epilepsy, autism, and schizophrenia (Uddin et al. 2018) with varying frequency. These phenotypically overlapped genomic regions are comprised of genes that are highly constraint and might be involved in regulating different pathways related to multiple phenotypes. NDD and CA are phenotypically distinct yet co-occur often among rare disorders. For example, there are genomic regions that have been reported from both NDD and CA cases such as 22q11.2 microdeletion syndrome (McDonald-McGinn et al. 2015), which in some cases develop congenital heart diseases as a primary phenotype. However, there are also cases of 22q11.2 microdeletion syndrome with no apparent congenital anomalies (Rozas et al. 2019). Therefore, it is interesting to identify these overlapping regions from different phenotypes and delineate the pathways as the constraint genes underlying these overlapping phenotypic co-morbidities are still largely unknown.

In this study, we used phenotypically characterized large NDD and CA cohort data to identify the genes within the overlapping genomic regions impacted by de novo and rare CNVs. By applying pathway analysis and using human developmental transcriptome data, we found these genes to be associated with altered neural connectivity and selective tissue formation. Identification of the shared pathogenic mechanisms between NDDs and CAs will assist in effective diagnosis and targeted therapeutics.

## Materials and methods

### Sample details

**Fig. 1:**
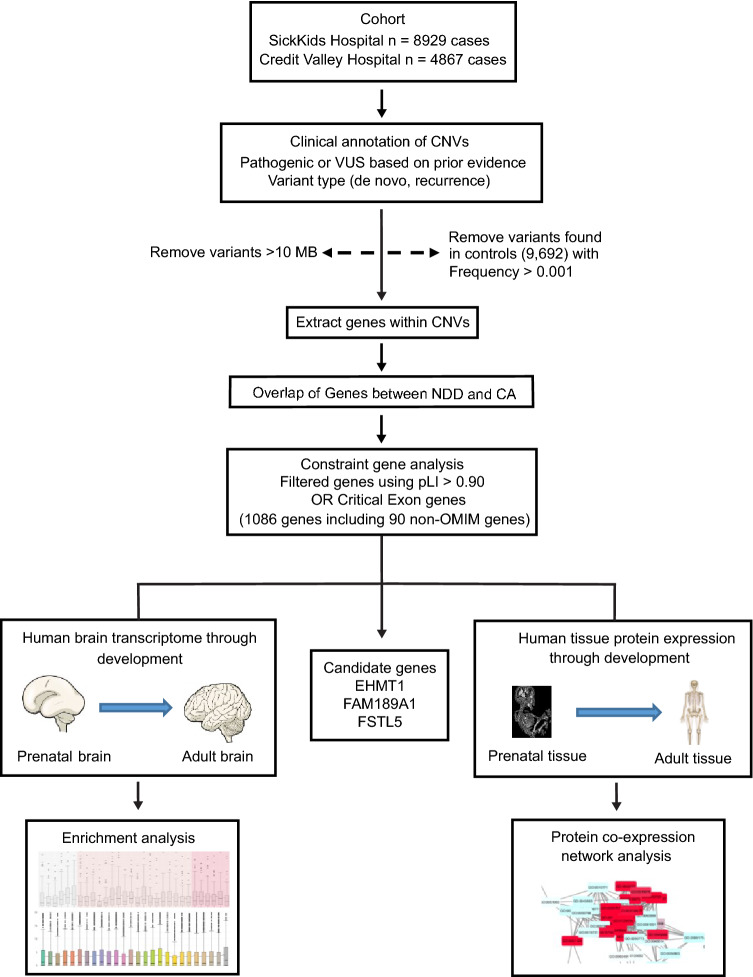
Schematic of study framework to identify and analyze overlapping (NDD and CA), constraint, candidate, and non-OMIM genes. Steps to identify candidate set of phenotypically relevant genes impacted by rare copy number variations (CNVs) (pathogenic/VUS; deletion/duplication) in neurodevelopmental disorder and congenital anomaly cases. Initial filtering was carried out with control variants (frequency) and constraint filtering using CE or pLI measures, and then performing enrichment in developmental human (prenatal, early childhood, and adult) brain transcriptome (RNA sequencing). *CNVs* copy number variations, *NDD* neurodevelopmental disorder, *VUS* variant of unknown significance

Clinical microarray data were collected from an NDD cohort (*n* = 10,620) (Uddin et al. 2016) with unrelated patients reported with phenotypes of autism spectrum disorder, language/speech delays, developmental delay, learning disability, mental retardation, seizures, or hypotonia. Our second cohort comprised of unrelated cases (*n* = 3176) consisting of a heterogeneous population carrying rare CAs with phenotypes of dysmorphic features, cleft palate, congenital heart defect, hypoplastic right heart, microcephaly, and (multiple) congenital anomalies (Uddin et al. 2016). These two cohorts were recruited from SickKids hospital (total cases *n* = 8929; NDD cases *n* = 7107; CA cases *n* = 1822) in Toronto, and Credit Valley Hospital (total cases *n* = 4867; NDD cases *n* = 3513; CA cases *n* = 1354) in Mississauga, respectively (Fig. 1, Table 1). Both cohorts consisted of a heterogenous population with similar geographic and socioeconomic backgrounds. Inclusion criteria for NDD samples comprised of the presence of any neurodevelopmental disorder as the primary phenotype which were documented by diagnostic behavior, phenotypes, and chromosomal microarray analysis. Regarding CAs cohort, the primary phenotype was reported as multiple congenital anomalies and congenital heart defects. There also exists the possibility that the CA patients might have some degree of NDD phenotype as a secondary manifestation that may have been under-reported.

**Table 1:** Demographics of the two cohorts specifying the breakdown in number of patients by phenotype, hospital, and gender

	SickKids Hospital	Credit Valley Hospital
Neurodevelopmental disorders		
Male	4862	2417
Female	2245	1096
Congenital anomalies		
Male	958	763
Female	865	590

### Chromosomal microarray analysis

A circular binary segmentation algorithm (Olshen et al. 2004) was applied on obtained clinical microarray data from both hospitals using International Standards for Cytogenomic Arrays ISCA 180 K comparative genomic hybridization array (aCGH) to detect large CNVs. To compare individual probe intensities, we used a pool of ten samples for reference. Each sample variant was annotated by employing numerous tools, including ANNOVAR (Wang et al. 2010) and Horizon platform of GenomeArc Analytics. The clinical laboratory geneticist manually annotated pathogenicity (pathogenic, likely pathogenic, variant of unknown significance (VUS), likely benign) of each CNV applying American College of Medical Genetics (ACMG) guidelines (Kearney et al. 2011). CNVs smaller than 10 Kb and larger than 10 Mb were excluded from analysis. The original dataset had de novo variant information, where parent DNA was accessible (Uddin et al. 2016).

### Control dataset

In this study, data from 9692 unrelated samples (Uddin et al. 2016) with no known psychiatric history have been used as population control (Fig. 1). This was collected from several major population-scale studies that utilized high-resolution microarray platforms. Illumina 1 M from the Study of Addiction Genetics and Environment (SAGE) (Bierut et al. 2010) and the Health, Aging, and Body Composition (HABC) (Coviello et al. 2012) assayed 4347 control samples; Illumina Omni 2.5 M from the Cooperative Health Research in the Region of Augsburg KORA projects (Verhoeven et al. 2013) and Collaborative Genetic Study of Nicotine Dependence (COGEND) (Bierut et al. 2007) assayed 2988 control samples; and Affymetrix 6.0 from the PopGen project (Krawczak et al. 2006) and the Ottawa Heart Institute (Stewart et al. 2009) assayed 2357 control samples. Using a high-resolution control will allow us to improve false positive calls from the ISCA low resolution case cohorts and will provide convincing association signals.

### Gene set curation and overlap analysis

**Fig. 2:**
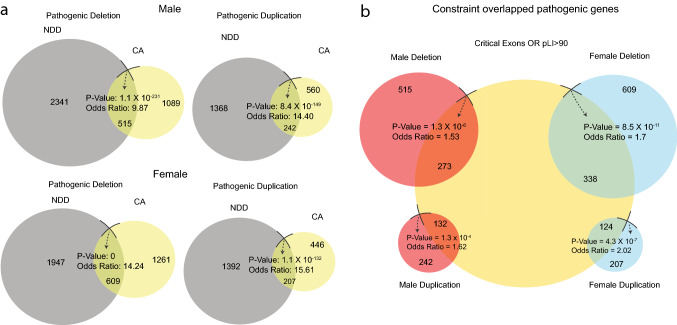
Diagrams displaying the significance of the overlap between neurodevelopmental disorder and congenital anomaly cases. **a** Venn diagram displaying the significance (FET: *P* value and Odds Ratio) of overlapped pathogenic gene lists between neurodevelopmental disorder (NDD) and congenital anomaly (CA) CNVs in males and females, before filtering with constraint measures (CE and pLI). **b** Venn diagram displaying the significance (FET, *P* value and Odds Ratio) of overlap between constraint (CE or pLI) gene list and the respective genes extracted from NDD and CA CNVs present in males and females, respectively. *FET* Fisher’s exact test

We used the GRCh37/hg19 build and unique coding sequence (CDS) ids for identifying regions of the DNA that encode for proteins, and removing repeats or duplicates, to analyze our data. CNVs were interpreted based on probable clinical significance or pathogenicity, variant type (deletion, duplication), inheritance (familial, de novo), gender (male, female), phenotype (NDD, CA), gene density and content (Additional file 1: Suppl. Fig. 1). First, we extracted the genes from the control dataset with frequency > 0.001 using the respective CDS ids, and similarly, we extracted the genes from the respective CNVs using the CDS ids. Subsequently, all gene overlaps between the control gene lists and patient gene lists were removed. In addition, the remaining genes extracted from CNVs based on gender, pathogenicity, and type were compiled (Fig. 2). We performed Fisher’s exact test (FET) using the R package (GeneOverlap) to measure statistical significance (*P* value < 0.05).

### Proteomic and multi-tissue transcriptome expression analysis

We used proteomic data from human protein expression studies at different developmental stages and expression data from multiple tissues to further analyze the genes that were extracted from NDD CNVs and had no overlap with genes from the CA CNVs. Proteomic and multi-tissue transcriptome datasets are described in detail in the following section.

#### Proteomic data analysis

To analyze protein expression levels at two developmental stages in human tissues, we used high-resolution genome-wide Fourier transform mass spectrometry data (downloaded from the Human Proteome Map) (Kim et al. 2014), including in-depth proteomic profiling of 30 histologically normal human samples: 7 fetal tissues (heart, liver, gut, ovary, testis, brain, and placenta), and 18 adult tissues (frontal cortex, spinal cord, retina, heart, liver, ovary, testis, lung, adrenal, gallbladder, pancreas, kidney, esophagus, colon, rectum, urinary bladder, and prostate), and 6 hematopoietic adult cells (B cells, CD4 cells, CD8 cells, NK cells, monocytes, and platelets) (Additional file 1: Suppl. Fig. 5) (Kim et al. 2014). For processing the data, fragmentation (high–high mode) was applied using the high-resolution Fourier transform mass spectrometers, identifying the proteins encoded by 17,294 genes, which accounts for 84% of annotated protein-coding human genes (Kim et al. 2014). For measuring protein expression, we used spectral counts per gene per sample. We performed Fisher’s exact *t*-test for the overlapped NDD and CA gene lists with CE or pLI enrichment using the R package (GeneOverlap) to measure statistical significance.

#### Multi-tissue transcriptome analysis

We measured expression levels (in triplicate) using Affymetrix GeneChip Human Exon 1.0 ST array (Gardina et al. 2006) and transcriptomes from cerebellum, breast, heart, liver, muscle, kidney, thyroid, pancreas, prostate, spleen, and testis, removing probes prone to multiple hybridizations. We used the Robust Multi-array Average (RMA) algorithm (Irizarry et al. 2003) to subtract the background signal and normalized the log2 expression values for each exon. Expression of 16,713 RefSeq genes was surveyed in all 11 tissues. A log2-transformed intensity threshold of ≥ 6 to define the expression (Kang et al. 2011) was used to detect 16,411 genes with at least one exon expressed in a tissue sample. Reads per kilobase of transcript per million (RPKM) was used as the expression unit for exons from the mapped reads (Additional file 1: Suppl. Fig. 6).

The CNVs chosen for proteomic and multi-tissue transcriptome data were genes from NDD pathogenic deletion CNVs that were not overlapped with CA gene list.

### Constraint gene analysis and data filtering

We have defined ‘constraint genes’ in our analysis if a gene present in both NDD and CA CNVs had a significant overlap with either critical exon (CE) or pLI ≥ 0.9 (probability of being Loss of Function intolerant). CE and pLI filtering methods are described in detail in the following section.

#### Spatiotemporal expression data from human brain and critical exons (CE)

**Fig. 3:**
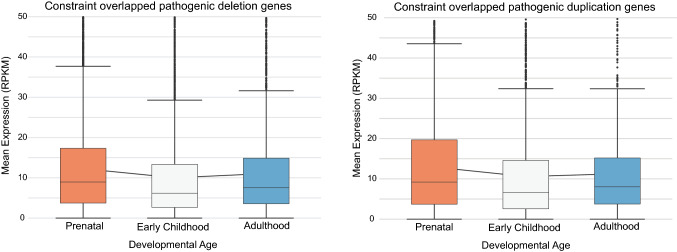
Developmental transcriptomics of constraint pathogenic genes. Boxplots displaying the expression of constraint overlapped (NDD and CA) genes extracted from pathogenic deletion and duplication CNVs in developmental brain transcriptome data of prenatal (8–37 weeks post-conception), early childhood (10 months to 15 years), and adult subjects (> 17 years) displayed on the x-axis. Y-axis represents normalized gene expression in Reads per kilobase per million (RPKM) units. Boxplots showing median, interquartile range (IQR) with whiskers adding IQR to the 1st and 3rd quartile, and the line connecting the three boxes is comparing the mean expression of the three developmental periods

Critical exons are highly expressed exons with low mutation burden. For this project, we have recalculated critical exon matrix based on our previous work (Uddin et al. 2016). For deleterious genes that harbor de novo mutations, critical exons were significantly enriched in individuals with ASD relative to their siblings without ASD (Uddin et al. 2014). We utilized these highly specific set of genes (critical exon genes) derived from computing exon level spatiotemporal RNA-seq expression of 388 tissue samples (derived from 42 different brain donors). RPKM was used as the expression unit for exons from the mapped reads. The selection of donors was made to include at least two sex and aged-matched donors, and each developmental period: prenatal (8–37 weeks post-conception), early childhood (10 months to 15 years), and adulthood (> 17 years). We derived the expression data of 16 brain regions within 3 developmental periods for each donor (Fig. 3 and Additional file 1: Suppl. Fig. 7). We used gnomAD to identify the non-synonymous rare (< 0.01 frequency) mutation burden. An exon is categorized as ‘critical exon’ if its expression is high (> 75th percentile) and gnomAD population non-synonymous mutation burden is low (< 75th percentile) compared to the entire dataset. A gene is considered a ‘critical exon gene’ if one or more exons were annotated as ‘critical exon’ for at least 50 RNA-seq brain samples.

#### pLI

As a second filtering criteria for constrained genes, we obtained pLI scores from Exac database to identify the tolerance of a susceptible gene to loss of function and pLI ≥ 0.9 are extremely LoF intolerant (Lek et al. 2016). We performed Fisher’s exact *t*-test for the overlapped gene lists (NDD and CA) with CE or pLI enrichment using the R package (GeneOverlap) to measure statistical significance.

### Pathway enrichment analysis

We performed the enrichment analysis of the most significant gene overlaps from the respective type of CNVs to determine the major pathways in which the constrained genes were expressed. We scanned the KEGG pathway database which comprises of an assembly of the up-to-date interactions, reactions, and relations of molecular networks (http://www.genome.jp/kegg/pathway.html) and GO database (http://geneontology.org/) to identify all the pathways in which five or more genes (from the constraint gene set) were expressed. Only the pathways having more than 50 genes and less than 1000 genes were considered for this analysis. We called a gene set enriched if it overlapped between our gene set and the KEGG-GO pathway database with significance (Fischer Exact Test (FET)). The pathways were identified by their unique KEGG ID and name. The significant pathways (*P* < 0.05) with a false discovery rate (FDR) < 0.01 were used to construct the pathway network map using Cytoscape (https://cytoscape.org/) for visualization.

## Results

### Overlapping genes extracted from de novo CNVs in NDD and CA cases

After analyzing a total of 13,796 patients from both cohorts, 195 patients (151 NDD cases (77% of de novo cases) and 44 CA cases (23% of de novo cases)) contained a total of 218 validated de novo CNVs from which 50 de novo CNVs overlapped between the NDD and CA cases (18% (31/170) of de novo NDD CNVs and 40% (19/48) of de novo CA CNVs have overlapping genomic regions impacting developmentally constraint genes) (Additional file 2: Suppl. Table 1). The phenotypes of the de novo CNVs that overlapped were developmental delay, multiple congenital anomalies, and autism (Additional file 1: Suppl. Fig. 4, Additional file 2: Suppl. Table 1). After filtering small (CNVs < 10 Kb) and large (CNVs ≥ 10 Mb) variants, 126 pathogenic CNVs (97 NDD and 29 CA) (Additional file 1: Suppl. Fig. 1b), and 80 VUS CNVs (65 NDD and 15 CA) were retained (Additional file 1: Suppl. Fig. 2a). Larger CNVs (size range of 1–5 Mb) were most prevalent compared to smaller CNVs (Range < 1 Mb). NDD pathogenic CNVs were present across 55 male and 36 female, and CA pathogenic CNVs across 15 male and 14 female cases.

Genes per de novo variants averaged mostly in the 1–50 kb range, comprising more than 70% of the number of genes in each respective exonic variant size category (Additional file 1: Suppl. Fig. 1b, Additional file 1: Suppl. Fig. 2a). After filtering out the genes from the de novo CNVs by overlapping with control gene set, we discovered 138 de novo genes to be impacted by at least one pathogenic deletion (*P* value = 2.87 × 10^–90^, OR = 14.69) in CNVs containing both NDD and CA cases. Similarly, significant overlap of 72 genes from the de novo pathogenic duplications (*P* value = 9.7 × 10^–62^, OR = 22.12) were found. The overlap of de novo VUS deletion gene set with control were not significant (*P* value = 0.14, OR = 6.41), so is the overlap of genes from de novo NDD and CA VUS duplications after filtering with control genes (*P* value = 1, OR = 0); therefore, we decided to provide two separate results: (1) unfiltered and (2) filtered using the controls and the pre-determined criteria. In the unfiltered analysis, there was a significant overlap of 13 de novo VUS deletion genes (*P* value = 5.65 × 10^–16^, OR = 38.67) between the NDD and CA cases but not among the duplications (*P* value = 0.08, OR = 2.51). Whereas after filtering, there was no significant overlap between the union of de novo VUS deletion gene sets (NDD and CA) (post-filtering with controls) and pLI and CE filters (as described in the methods) (*P* value = 0.43, OR = infinite). Similarly, there was no significance between the overlap of genes from de novo NDD and CA VUS duplications after filtering (*P* value = 1, OR = 0).

#### Constraint overlapping genes from the pathogenic NDD and CA de novo CNVs

After constraint gene filtering analysis, significant overlap was observed for 79 (10.1% non-OMIM entries) de novo pathogenic deletion CNV affected genes (*P* value = 0.01, OR = 1.58) and 45 (13.3% non-OMIM entries) de novo pathogenic duplication affected genes (*P* value = 0.01, OR = 1.97) (Additional file 2: Suppl. Table 2a/2b).

### Overlapping genes in NDD and CA CNVs across gender (pre-filtering)

#### Male

Five hundred and fifteen genes with at least 1 exon impacted by pathogenic deletion CNVs in male show significant (*P* value = 1.1 × 10^–231^, OR = 9.87) overlap between the genes from NDD and CA cases (Fig. 2a). Similarly, significant overlap was also observed for 242 genes from pathogenic duplications in male (*P* value = 8.4 × 10^–149^, OR = 14.40) (Fig. 2a). The VUS gene lists had 112 intersected genes from (deletions that showed significant (*P* value = 1.2 × 10^–58^, OR = 9.53) the overlap between the NDD and CA cases in male (Additional file 1: Suppl. Figure 3a). Significant overlap was also observed for 320 VUS duplications in male (*P* value = 1.1 × 10^–99^, OR = 4.47) (Additional file 1: Suppl. Figure 3a).

#### Female

Six hundred and nine genes with at least 1 exon impacted by pathogenic deletions in female show significant (*P* value < 0, OR = 14.24) overlap from NDD and CA cases (Fig. 2a). Similarly, significant overlap was also observed for 207 genes within pathogenic duplication CNVs in female (*P* value = 1.1 × 10^–132^, OR = 15.61) (Fig. 2a). The overlapped VUS gene lists had 64 intersected genes with at least one exon impacted by deletions (*P* value = 5.8 × 10^–31^, OR = 7.60) between the NDD and CA cases in female (Additional file 1: Suppl. Figure 3a). Significant overlap was also observed for 168 genes within VUS duplications in female (*P* value = 9.8 × 10^–42^, OR = 4.06) (Additional file 1: Suppl. Fig. 3a).

### Constraint genes within the overlapped NDD and CA cases across gender (post-filtering)

#### Male

After applying CE and pLI constraint gene thresholds (detailed in Methods), 273 overlapped genes with at least 1 exon impacted by pathogenic deletions were found in male (*P* value = 1.30 × 10^–6^, OR = 1.53) with both NDD and CA cases (Fig. 2b). Significant overlap was also observed for 132 constraint genes impacted by pathogenic duplications in male (*P* value = 1.3 × 10^–4^, OR = 1.62) (Fig. 2b). After constraint filtering of the overlapped VUS gene lists, 46 genes with at least 1 exon impacted by deletions showed no significant (*P* value = 0.67, OR = 0.93) overlap between the NDD and CA cases (Additional file 1: Suppl. Fig. 3b). Significant overlap was observed for 320 constraint genes impacted by VUS duplications (*P* value = 6.9 × 10^–4^, OR = 1.37) (Additional file 1: Suppl. Figure 3b).

#### Female

After filtering the constraint overlapped gene lists, 338 genes with at least 1 exon impacted by pathogenic (OR = 1.69) deletions showed significant (*P* value = 8.5 × 10^–11^) overlap between the genes from NDD and CA cases in female (Fig. 2b). Similarly, significant overlap was also observed for 124 intersected genes from constraint pathogenic duplications (*P* value = 4.3 × 10^–7^, OR = 2.02) (Fig. 2b). After constraint filtering of the overlapped VUS gene lists, 64 genes with at least 1 exon impacted by deletions showed no significant (*P* value = 0.11, OR = 1.84) overlap between the NDD and CA cases (Additional file 1: Suppl. Figure 3b). Significant overlap was observed for 89 intersected genes from constraint VUS duplications (*P* value = 4.5 × 10^–3^, OR = 1.52) (Additional file 1: Suppl. Fig. 3b).

#### X-chromosome analysis

Among the CNVs that impacted constraint overlapped gene lists the X-chromosome were pathogenic duplication and VUS duplication CNVs found in both males and females (Additional File 2: Suppl. Table 3). There were 11 pathogenic duplication CNVs (7 NDD, 4 CA CNVs) in males and 4 pathogenic duplication CNVs (2 NDD, 2 CA CNVs) in females, and 13 VUS duplication CNVs (7 NDD, 6 CA CNVs) in males and 4 VUS duplication CNVs (1 NDD, 3 CA CNVs) in females (Additional File 2: Suppl. Table 3). There were no details available on the specific phenotypes other than the broad category.

### Expression of constraint NDD genes in developmental brain and multi-tissue transcriptome and proteome

Analysis of the developmental brain transcriptome data demonstrated prenatal expression to be the highest for both constraint overlapped pathogenic deletion (*P* value = 4.95 × 10^–6^) and duplication genes (*P* value = 0.01), followed by adulthood, and early childhood, respectively (Fig. 3). Differential proteomic tissue expression demonstrated that the adult testis and adult retina have the highest expression in pathogenic deletion and duplication genes, respectively (Additional file 1: Suppl. Fig. 5). Differential transcriptomic tissue expression was non-specific for NDD pathogenic deletion and duplication genes (Additional file 1: Suppl. Fig. 6).

### Candidate gene-specific mutation data

We identified 1086 constrained genes whose mutation might contribute to NDD and CA phenotypes. Three unique candidate genes, *EHMT1*, *FAM189A1*, and *FSTL5*, were chosen, with the former selected from the overlapped pathogenic deletion genes list, identified in the respective CNVs less than 1 Mb. *EHMT1* was found in four deletion CNVs (the highest frequency from our CNV data) and this CNV was considered pathogenic with respect to both NDD and CA phenotype (Frega et al. 2019). The remaining two novel candidate genes were selected from the significant overlapped gene lists after filtering with CE and pLI > 0.90 that contained no Online Mendelian Inheritance in Man (OMIM) entries (total of 90 unique genes impacted by 90 CNVs) (Additional file 2: Suppl. Table 2a) and had the highest number of gene-specific CNVs in the literature. We reviewed additional cohorts in DECIPHER (DatabasE of genomiC varIation and Phenotype in Humans using Ensembl Resources) and publications (Pubmed) (Additional file 2: Suppl. Table 4).

### Candidate gene: *EHMT1*

**Fig. 4:**
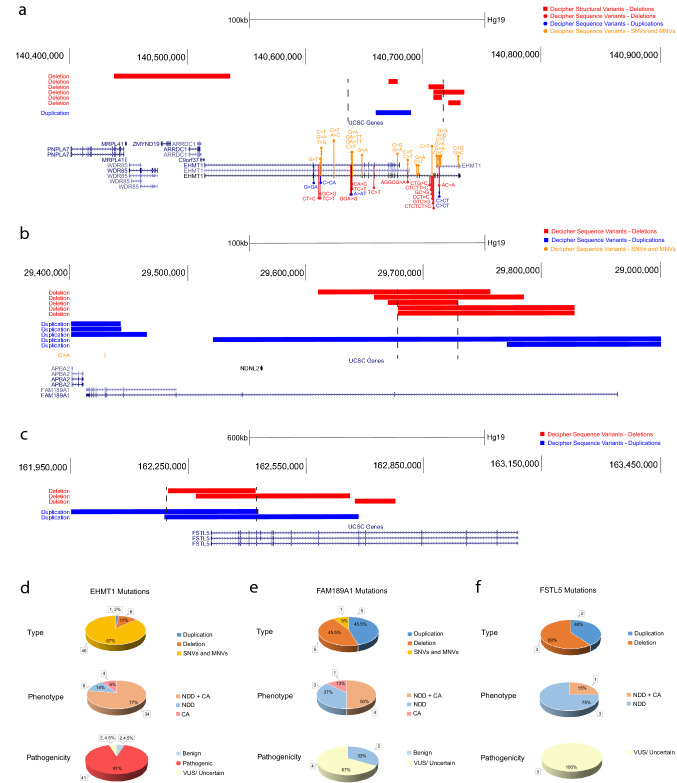
Mutational landscape in candidate genes *EHMT1*, *FAM189A1*, and *FSTL5*. **a** The breakpoints of pathogenic deletion (red) and duplication (blue) variants impacting *EHMT1,* and nearby genes reported in DECIPHER. **b** The breakpoints of pathogenic deletion (red) and duplication (blue) variants impacting *FAM189A1,* and nearby genes reported in DECIPHER. **c** The breakpoints of pathogenic deletion (red) and duplication (blue) variants impacting *FSTL5* and nearby genes reported in DECIPHER. **d** The pie charts describe the count and percentage of *EHMT1* variant mutations reported in DECIPHER with the following variables: type, phenotype, and pathogenicity. **e** The pie charts describe the count and percentage of *FAM189A1* variant mutations reported in DECIPHER. **f** The pie charts describe the count and percentage of *FSTL5* variant mutations reported in DECIPHER. DECIPHER*,* DatabasE of genomiC varIation and Phenotype in Humans using Ensembl Resources; SNV, single-nucleotide variant; MNV, multi-nucleotide variant

Pathogenic deletions in our cohort within chromosome region 9q34.3 affected the gene, euchromatic histone methyltransferase 1 (*EHMT1*) (Fig. 4a). The EHMT1 protein is known to control brown adipose cell fate and is an essential brown adipose tissue (BAT)-enriched lysine methyltransferase in the PRDM16 transcriptional complex (Ohno et al. 2013). From clinical cohorts (DECIPHER), we found enrichment of CNVs less than 10 Kb affecting *EHMT1* among cases (6 deletions, 1 triplication, and 46 single-nucleotide variants, including 33 de novo) (Fig. 4a/d) (Additional file 2: Suppl. Table 4a). Schaefer et al. reported that knock-out EHMT1 -/- mice decreased euchromatic H3K9 methylation in the forebrain and upregulation of neuronal and non-neuronal genes, especially affecting those involved in developmental stage-dependent gene expression (Schaefer et al. 2009). Moreover, the KO mice displayed defects in learning and memory, and demonstrated *EHMT1* to be a key regulator of transcriptional homeostasis of cognition and adaptive behavior (Schaefer et al. 2009).

### Candidate gene: *FAM1891A*

After enriching the overlapped gene lists with critical exons and pLI, we formulated a non-OMIM gene list from which *FAM189A1* (family with sequence similarity 189 member A1) (Fig. 4b/e) was the only de novo gene that contained at least two gene-specific CNVs (a total of five deletions) (Fig. 4b/e). It was also present in the de novo pathogenic deletion list (Additional file 2: Suppl. Table 2b), and in both male and female overlapped gene lists (Additional file 2: Suppl. Table 2c and d). In clinical cohorts (DECIPHER), we found enrichment of CNVs less than 1 Mb affecting *FAM189A1* among cases (five deletions and five duplications) (Additional file 2: Suppl. Table 4b). The gene is expressed in the pancreatic tissue (specialized epithelial cells) and thyroid gland with single-cell type specificity in the neuronal cells of the brain (Human Protein Atlas (http://proteinatlas.org)) (Uhlén et al. 2015). In a study conducted by Murray et al. on genome-wide association between individuals with life-threatening arrhythmia and normal controls in the span of at least 3 years (Murray et al. 2012), the highest *P* value of 5.0 × 10^−6^ and odds ratio of 2.02 were located in the gene *FAM189A1*.

### Candidate gene: *FSTL5*

*FSTL5* (Follistatin-like 5) is the other non-OMIM entry candidate gene within the overlapped gene lists that are enriched with critical exons and pLI > 0.9 (Additional file 2: Suppl. Table 2a). It was only identified in the female pathogenic deletion overlapped gene lists. From published data and in clinical cohorts (DECIPHER), we found enrichment of CNVs less than 1 Mb affecting *FSTL5* among cases (three deletions and two duplications) (Fig. 4c/f) (Additional file 2: Suppl. Table 4c). FSTL5 is hypothesized to be an extracellular protein with roles in enabling calcium ion binding activity and cell differentiation [provided by Alliance of Genome Resources, Apr 2022 (Agapite et al. 2020)]. The gene is expressed in retina and brain according to the Human Protein Atlas (http://proteinatlas.org) (Uhlén et al. 2015). Studies have shown an array of functions for FSTL5 in the human body, ranging from inhibiting the progression of hepatocellular carcinoma (Zhang et al. 2015, 2020; Li et al. 2018) to being a marker of poor prognosis in Non-WNT/Non-SHH medulloblastoma (Remke et al. 2011).

### Pathways enriched in NDD and CA gene sets

**Fig. 5:**
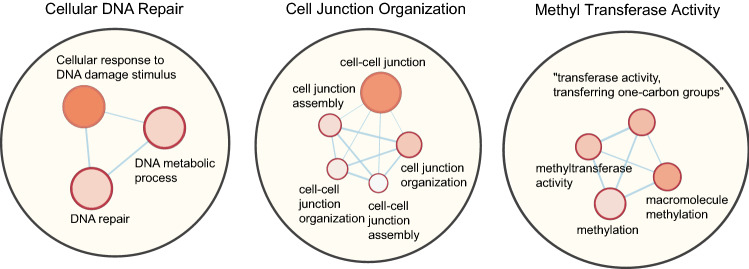
Pathway network map of constraint overlapped (NDD and CA) genes. Pathway network analysis of constraint overlapped genes from de novo pathogenic deletion CNVs enriched clusters drawn using Cytoscape. The analysis of overlapped loss pathogenic genes with significant pathways (*P* value < 0.05) with a false-discovery rate (FDR) < 0.01. The color gradient and size of the node represented the *P* value and odds ratio, respectively

The genes in constraint overlapped (NDD and CA) de novo pathogenic deletion CNVs are enriched in important pathways such as cellular DNA repair, cellular junction organization, and methyl transferase activity (Fig. 5). NDD pathogenic deletion genes were enriched in biological pathways that include transmembrane ion transport, photoreceptor cilium activity, and organ system development (Additional file 1: Suppl. Fig. 8). However, the constraint genes in overlapped pathogenic deletion CNVs were significantly enriched in chemical synaptic transmission, catabolic activity, morphogenesis and differentiation (Additional file 1: Suppl. Fig. 9). These enriched pathways demonstrate the involvement of the overlapped genes in both NDD- and CA-related pathways.

## Discussion

The complex molecular interaction of genes may underly the phenotypic heterogeneity that may impact various developmental pathways [37]. In this study, we have used two large cohorts of NDD and CA patients to identify de novo variants and their associated constraint genes. We identified a core set of overlapping constraint genes that can help explain the complex molecular etiology of NDD and CA. Our result shows that these constraint genes (i) are significantly impacted by de novo pathogenic CNVs reported in both NDD and CA; (ii) are highly upregulated in prenatal stage of brain; (iii) are involved in developmental cellular pathways; and (iv) provide a list of candidate genes that may not be captured from individual cohort analysis (NDD or CA), rather captured in combined analysis.

The simultaneous presence of both phenotypes of NDDs and CAs has been described recently. Of significance, Fregaue et al. (2016) reported that proximal deletions of 1p36 or haploinsufficiency of the *RERE* gene, found in 10 subjects were strongly associated with the onset of both NDD and CA phenotypes, and this was also observed in RERE-deficient mice and zebrafish. Furthermore, Jordan et al. studied nine individuals with NEBDEH that had partial deletions or deleterious sequence variants in *RERE* (Jordan et al. 2018). CHARGE syndrome, a differential to RERE-related disorders, indicated to involve both NDDs and CAs, is reported to be caused by de novo mutations in the *CHD7* gene with a prevalence of 1 in 10,000 births (Jordan et al. 2018). Clinical features include coloboma, heart defects, choanal atresia, retarded growth and development, genital abnormalities, ear anomalies, and distinguishing features from RERE-related disorders are the presence of semicircular canal defects or tracheoesophageal fistulas in CHARGE syndrome patients (Hsu et al. 2014).

Our study offers an initial comparison of the two sexes to detect genes and variants in NDD and CA phenotypes for all the autosomes and X-chromosome. By assessing 13,796 sequenced patients, we identified 217 CNVs with enrichment of de novo variants in patients with NDDs and CAs, irrespective of gender. Comparatively, de novo mutations in these variants were greater in males than in females. Male de novo pathogenic deletion variants contained nine more CNVs larger than 5 Mb compared to females and that may explain the increased prevalence of de novo CNVs in males.

Comparing all sets of gene overlaps (from male and female CNVs), we identified the NDD and CA overlap of genes extracted from pathogenic deletion CNVs in female to be the most significant (*P* value = 0) and the overlap of genes extracted from pathogenic duplication CNVs in female to have the highest odds ratio (OR = 15.61). Similarly, among the overlapped genes from pathogenic CNVs that underwent constraint filtering using CE or pLI, the gene overlap from the pathogenic deletion variants in female were the most significant (*P* value = 8.5 × 10^–11^) and the gene overlap from pathogenic duplication CNVs in female held the highest odds ratio (OR = 2.02).

Constraint genes are highly upregulated in prenatal period which shows their importance in early neurogenesis and organ development. Out of the constraint overlapped genes (1086 genes), we shortlisted three genes, one with possible studied pleiotropism of NDDs and CAs: *EHMT1*, which causes Kleefstra syndrome, known to harbor heterozygous intragenic *EHMT1* pathogenic variants from heterozygous deletions at chromosome 9q34.3 (Yatsenko et al. 2009; Willemsen et al. 2012). This syndrome involves NDD characteristics of childhood atonia, autistic-like features, intellectual disability, and CA characteristics of distinctive facial features (Cormier-Daire et al. 2003; Stewart et al. 2004; Kleefstra et al. 2005; Yatsenko et al. 2005), heart defects, renal/urologic defects, and genital defects in males among others. It is reported that both genders are equally affected and with some indication of genotype–phenotype correlation in 9q34.3 deletions affected by pathogenic variants that are smaller in size (< 1 Mb) (Yatsenko et al. 2009; Kleefstra et al. 2009; Willemsen et al. 2012). However, the grouping of several haplo-insufficient genes generates a pathological phenotype, and a direct causal relationship of phenotype to an individual gene cannot be ascertained. The other candidate genes, *FAM189A1* and *FSTL5*, were selected from the non-OMIM gene list that was curated from the constraint filtered 1089 candidate genes. Our study suggests the possible roles of these genes in NDDs and CAs that have no OMIM entries.

Establishing genotype and phenotype correlation is complex (Uddin et al. 2019), especially for constraint genes that are reported in multiple distinct phenotypes (Woodbury-Smith et al. 2017a, b). Future development of artificial intelligence coupled with deep phenotypic information might improve the delineation of constraint genes that may underly the etiology of NDD and CA. We have demonstrated the multi-faceted use of different types of molecular data from the human brain tissue to interpret and identify candidate genes for NDD and CA disorders, from pathogenic variants and VUS. One of the limitations of our study might be the under-reporting of phenotypes between the cases of the two cohorts, as some of the CA cases might have later developed NDD symptoms which cannot be captured in a retrospective cohort without reevaluating the patient status. Our assessable approach considering the reported phenotypes enables the indexing of genes affected by respective CNVs for a possible role in neurodevelopmental disorders and congenital anomalies.

It may be possible to develop effective targeted treatment with single-cell analysis of overlapping NDD and CA genes in the same way that the identification of cancer subtypes could enable and guide cancer treatments (Parsons et al. 2008; Chapman et al. 2011; Herbst et al. 2014). This study is a proof-of-concept for how massive amounts of CNV and transcriptomic data can be used to expand existing knowledge and bring precision medicine to treating NDD and CA cases with overlapping genes and CNVs. Further studies to understand the functional regulation of the candidate genes may help in targeted therapeutics and timely interventions throughout development to mitigate the effects of different genomic alterations.

## Conclusion

We have incorporated multi-dimensional transcriptome data from different sources to understand the genetic overlap of NDDs and CAs. We observed that those different mutations may be implicated in a molecular subtype of NDD and CA. By applying an integrative framework, we examined the convergence of clinical mutations onto specific disease-related pathways. The comprehensive analytical framework in our work can be utilized to uncover functional elements for other genetic diseases, enhancing their risk assessment. The overlap of molecular subtypes of NDD and CA risk genes to brain tissue cell types and pathways will be vital for the future development of effective combined diagnosis of NDD and CA and aid in therapeutics.

## Supplementary Information

Below is the link to the electronic supplementary material.

**Suppl. Fig. 1:** Descriptive statistics of CNV data of pathogenic variants in cohorts. **a** The CNV size distribution based on phenotype and gender compared to the whole data. Bars indicate the total number of CNVs in each classification. **b** The percentage of male and female cases in the cohort impacted by *de novo* pathogenic deletion and duplication variants less than 10 Mb, with the CNV size distribution and number of genes per CNV. **C** The CNVs of 0 kb to 10 Mb were classified based on the percentage of male and female cases in the cohort impacted by pathogenic deletion and duplication variants, with the CNV size distribution and number of genes per CNV. Of all samples assayed, 0.0038% carried a pathogenic deletion, 0.0016% a pathogenic duplication (PDF 769 KB)

**Suppl. Fig. 2**: Ascertainment and description of variants of uncertain significance (VUS) in cohorts. **a** The percentage of male and female cases in the cohort impacted by *de novo* VUS deletion and duplication variants less than 10 Mb, with the CNV size distribution and number of genes per CNV. **b** The CNVs of 0 kb to 10 Mb were classified based on the percentage of male and female cases in the cohort impacted by VUS deletion and duplication variants, with the CNV size distribution and number of genes per CNV. Of all samples assayed, 0.0177% were VUS deletion and 0.0320% a VUS duplication. VUS, variants of uncertain significance; CNV, copy number variant (PDF 673 KB)

**Suppl. Fig. 3**: Overlap of VUS between neurodevelopmental disorder and congenital anomaly cases. **a** Venn diagram displaying the significance (FET; P-value and Odds Ratio) of overlapped VUS gene lists between neurodevelopmental disorder (NDD) and congenital anomaly (CA) CNVs in males and females, before filtering with constraint measures (CE OR pLI). **b** Venn diagram displaying the significance (FET, P-value and Odds Ratio) of the overlap between constraint CE or pLI gene sets and the respective genes extracted from NDD and CA VUS CNVs present in males and females, respectively (PDF 1140 KB)

**Suppl. Fig. 4**: Overlapped *de novo *CNVs phenotypes. Pie chart displaying the count and percentage of characteristic phenotypes of the CNVs that contained constraint overlapped gene lists (filtered with CE OR pLI) between neurodevelopmental disorder (NDD) and congenital anomaly (CA) CNVs. The inner pie chart are CNVs from female cases and the outer pie chart are CNVs from male cases. The three phenotypes seen in descending order of prevalence are developmental delay (Total number: 30 CNVs (60% of overlapped *de novo *CNVs phenotypes), multiple congenital anomalies (Total number: 19 CNVs (38% of overlapped *de novo *CNVs phenotypes)), and autism (Total number: 1 CNV (2% of overlapped *de novo *CNVs phenotypes)) (PDF 373 KB)

**Suppl. Fig. 5**: Expression of NDD pathogenic deletion and duplication genes at different developmental stages across tissues. Boxplots displaying the protein expression levels of genes extracted from NDD CNVs but had no overlap with genes from the CA CNVs at two developmental stages in human tissues (Fetal and Adult) by using high-resolution genome-wide Fourier-transform mass spectrometry data containing in-depth proteomic profiling of 30 histologically normal human samples. Boxplots showing median, interquartile range (IQR) with whiskers adding IQR to the 1st and 3rd quartile, and the line connecting the boxes is comparing the mean expression of the different tissues. Y-axis represents normalised protein expression in spectral counts per gene per sample (SCGS). SCGS, spectral counts per gene per sample (PDF 782 KB)

**Suppl. Fig. 6**: Multi-tissue transcriptome expression analysis of genes from NDD CNVs. Boxplots displaying the expression in multi-tissue transcriptome analysis of genes extracted from NDD CNVs but had no overlap with genes from the CA CNVs. For the multiple tissue expression analysis, we used transcriptomes from 11 normal human tissues (cerebellum, breast, heart, liver, muscle, kidney, thyroid, pancreas, prostate, spleen, and testis) and measured expression levels (in triplicate) displayed in the X-axis. Boxplots showing median, interquartile range (IQR) with whiskers adding IQR to the 1st and 3rd quartile, and the line connecting the boxes is comparing the mean expression of the different tissues. Y-axis represents normalised gene expression in reads per kilobase per million (RPKM) units (PDF 430 KB)

**Suppl. Fig. 7**: Spatiotemporal association analysis of critical exons in constraint genes. Boxplots displaying the spatiotemporal transcriptome data of constraint overlapped genes extracted from pathogenic deletion and duplication CNVs. Boxplots showing median, interquartile range (IQR) with whiskers adding IQR to the 1st and 3rd quartile, and the line connecting the boxes is comparing the mean expression of the different tissues. Y-axis represents normalised gene expression in reads per kilobase per million (RPKM) units (PDF 687 KB)

**Suppl. Fig. 8**: Pathway network map of genes extracted from NDD pathogenic deletion CNVs. Pathway network analysis displaying enriched pathway clusters of genes from NDD pathogenic deletion CNVs that were attained by removing the significant gene overlap with the genes from CA pathogenic deletion CNVs, drawn using Cytoscape. The analysis of significant overlapped loss pathogenic genes with significant pathways (P-value < 0.05) with a false discovery rate (FDR) < 0.01. The color gradient and size of the node represented the P-value and odds ratio, respectively (PDF 527 KB)

**Suppl. Fig. 9**: Pathway network map of constraint overlapped genes from pathogenic deletion CNVs. Pathway network analysis in constraint overlapped genes from pathogenic deletion CNVs, drawn using Cytoscape. The analysis of significant overlapped loss pathogenic genes with significant pathways (P-value < 0.05) with a false discovery rate (FDR) < 0.01. The color gradient and size of the node represented the P-value and odds ratio, respectively (PDF 5206 KB)

**Suppl. Table 1**: *De novo* NDD and CA CNVs containing overlapping genes. This table lists out the CNVs that contain the genes extracted from *de novo* NDD and CA CNVs, that were overlapped using the respective unique coding sequence (CDS) ids**. **We performed Fisher’s exact test (FET) using the R package (GeneOverlap) to measure statistical significance (*P* value < 0.05) (XLSX 124 KB)

**Suppl. Table 2**: Candidate constraint overlapped gene lists from NDD and CA cases. **a **Candidate constraint overlapped genes from NDD and CA CNVs including non-OMIM genes. **b **Candidate constraint overlapped genes from *de novo* pathogenic NDD and CA CNVs. **c **Candidate constraint overlapped genes from NDD and CA pathogenic and VUS CNVs in females. **d **Candidate constraint overlapped genes from NDD and CA pathogenic and VUS CNVs in males (XLSX 124 KB)

**Suppl. Table 3**: Description of gender-specific variants reported on the X-chromosome from constraint overlapped gene lists. **a **Description of pathogenic duplication CNVs of male NDD and CA patients involving constraint overlapped genes on the X-chromosome. **b** Description of pathogenic duplication CNVs of female NDD and CA patients involving constraint overlapped genes on the X-chromosome. **c** Description of VUS duplication CNVs of male NDD and CA patients involving constraint overlapped genes on the X-chromosome.** d **Description of VUS duplication CNVs of female NDD and CA patients involving constraint overlapped genes on the X-chromosome (XLSX 74 KB)

**Suppl. Table 4**: Description of variants reported in DECIPHER of candidate genes *EHMT1*, *FAM189A1*, and *FSTL5*. **a **Description of variants reported in DECIPHER of candidate gene *EHMT1*. Locations were in GRCh38 and converted to hg19. **b** Description of variants reported in DECIPHER of candidate gene *FAM189A1*. **c** Description of variants reported in DECIPHER of candidate gene *FSTL5* (XLSX 19 KB)

## Data Availability

The datasets supporting the conclusions of this article are included within the article (and its Additional files).
