# Supplementary figures and images for: Overlapping pathogenic de novo CNVs in neurodevelopmental disorders and congenital anomalies impacting constraint genes regulating early development
Source: Hum Genet. 2022 Nov 16;142(8):1201–13. doi: 10.1007/s00439-022-02482-5 (PMC10449996; doi:10.1007/s00439-022-02482-5)

a

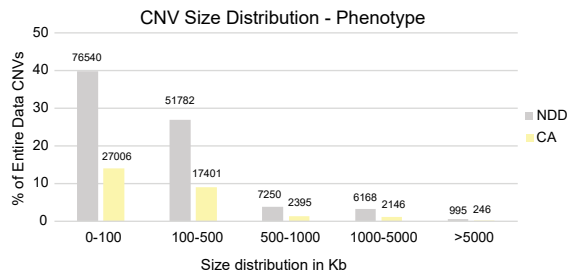

**CNV Size Distribution - Gender**

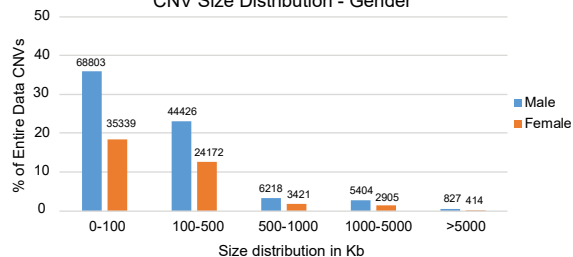

b

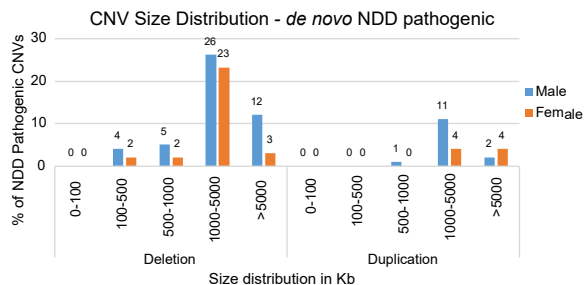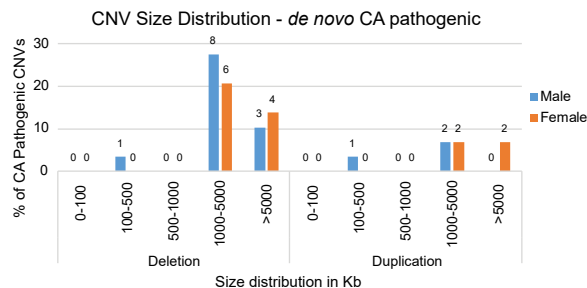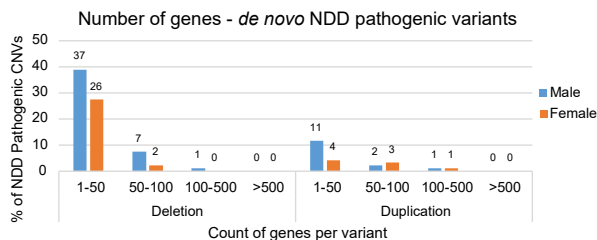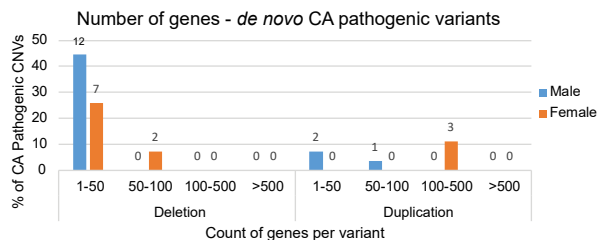

c

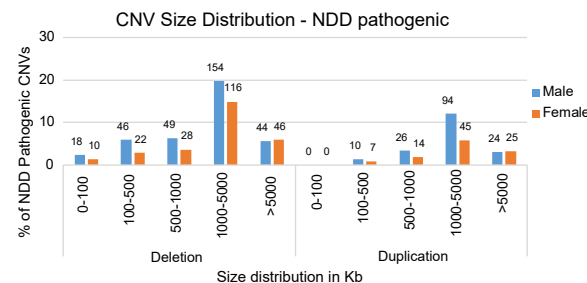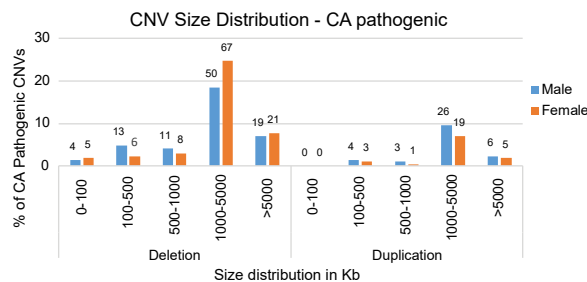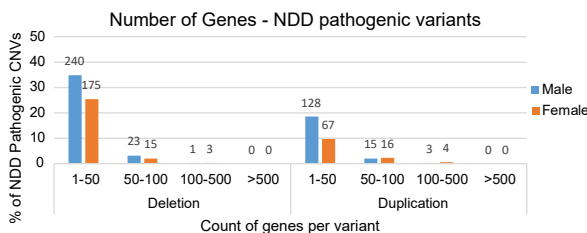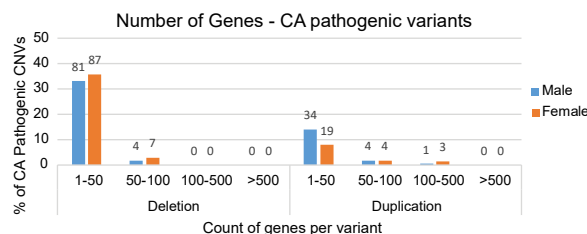

Supplement: Supplementary file 1 — Suppl. Fig. 1: Descriptive statistics of CNV data of pathogenic variants in cohorts. a The CNV size distribution based on phenotype and gender compared to the whole data. Bars indicate the total number of CNVs in each classification. b The percentage of male and female cases in the cohort impacted by de novo pathogenic deletion and duplication variants less than 10 Mb, with the CNV size distribution and number of genes per CNV. C The CNVs of 0 kb to 10 Mb were classified based on the percentage of male and female cases in the cohort impacted by pathogenic deletion and duplication variants, with the CNV size distribution and number of genes per CNV. Of all samples assayed, 0.0038% carried a pathogenic deletion, 0.0016% a pathogenic duplication (PDF 769 KB) [file 439_2022_2482_MOESM1_ESM.pdf]

a

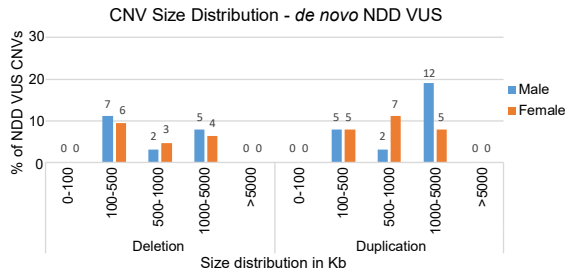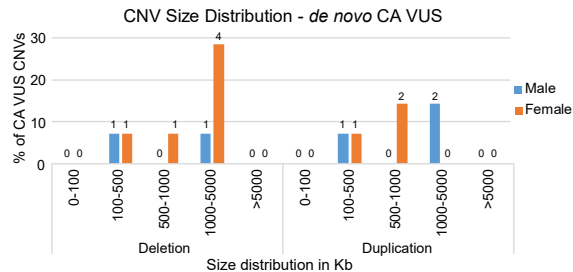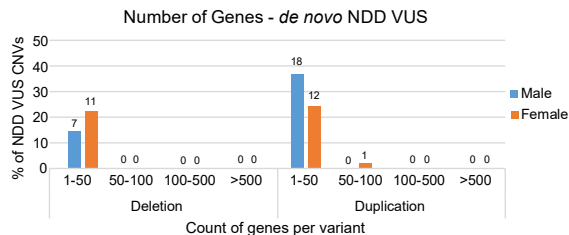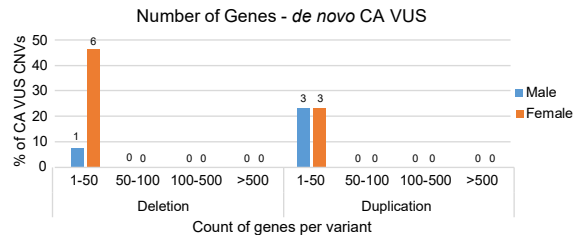

b

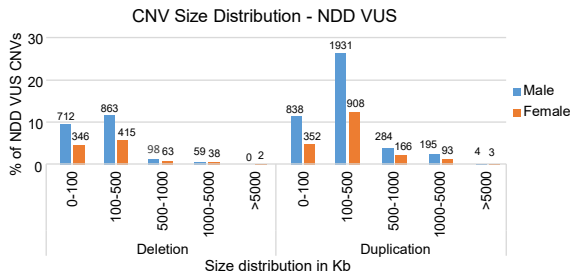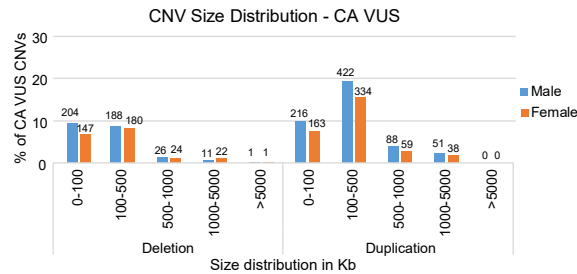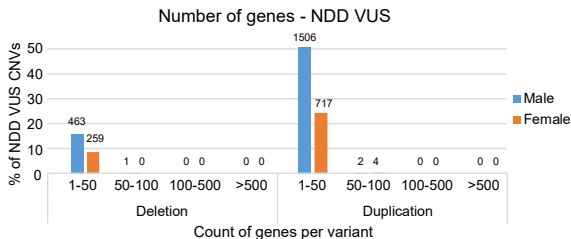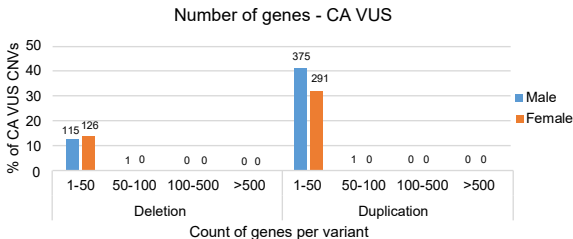

Supplement: Supplementary file 2 — Suppl. Fig. 2: Ascertainment and description of variants of uncertain significance (VUS) in cohorts. a The percentage of male and female cases in the cohort impacted by de novo VUS deletion and duplication variants less than 10 Mb, with the CNV size distribution and number of genes per CNV. b The CNVs of 0 kb to 10 Mb were classified based on the percentage of male and female cases in the cohort impacted by VUS deletion and duplication variants, with the CNV size distribution and number of genes per CNV. Of all samples assayed, 0.0177% were VUS deletion and 0.0320% a VUS duplication. VUS, variants of uncertain significance; CNV, copy number variant (PDF 673 KB) [file 439_2022_2482_MOESM2_ESM.pdf]

**a**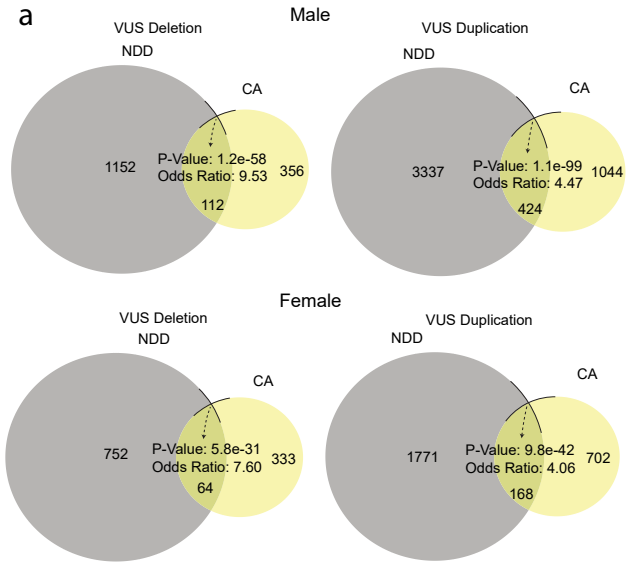**b**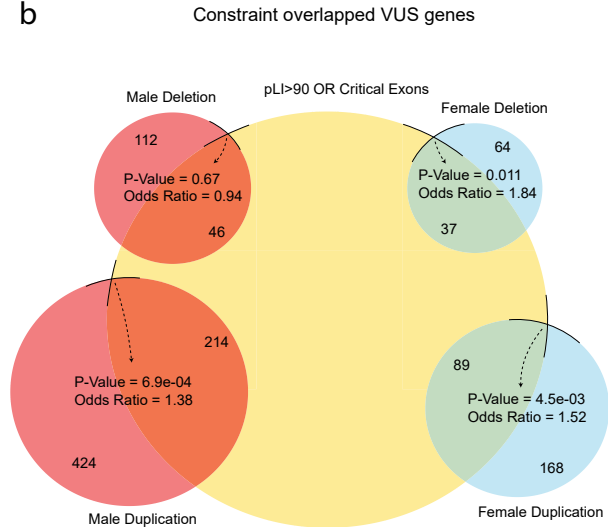

Supplement: Supplementary file 3 — Suppl. Fig. 3: Overlap of VUS between neurodevelopmental disorder and congenital anomaly cases. a Venn diagram displaying the significance (FET; P-value and Odds Ratio) of overlapped VUS gene lists between neurodevelopmental disorder (NDD) and congenital anomaly (CA) CNVs in males and females, before filtering with constraint measures (CE OR pLI). b Venn diagram displaying the significance (FET, P-value and Odds Ratio) of the overlap between constraint CE or pLI gene sets and the respective genes extracted from NDD and CA VUS CNVs present in males and females, respectively (PDF 1140 KB) [file 439_2022_2482_MOESM3_ESM.pdf]

# NDD pathogenic deletion genes

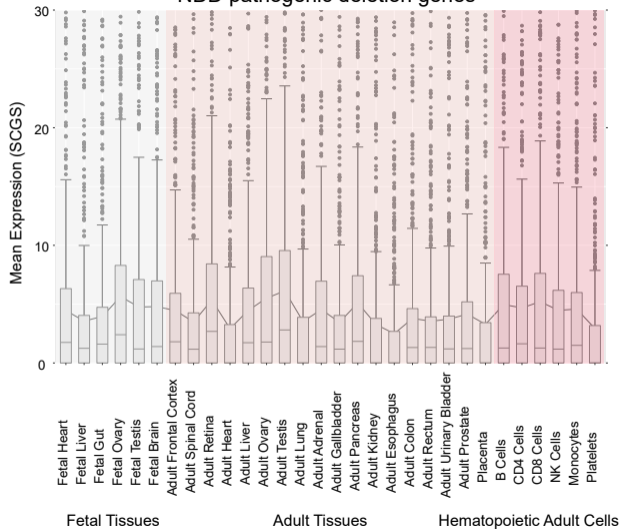

# NDD pathogenic duplication genes

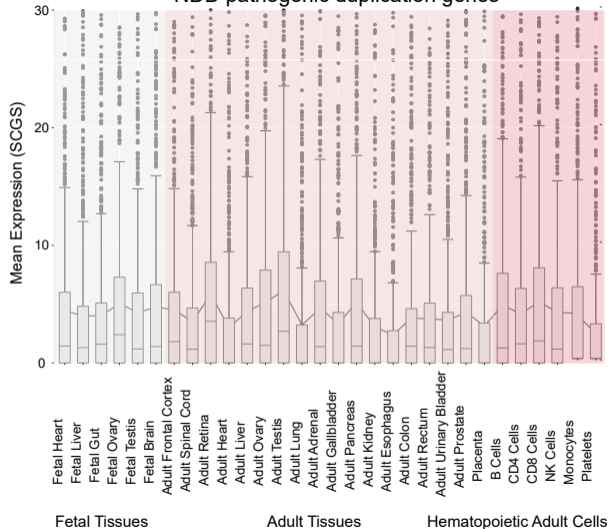

Supplement: Supplementary file 5 — Suppl. Fig. 5: Expression of NDD pathogenic deletion and duplication genes at different developmental stages across tissues. Boxplots displaying the protein expression levels of genes extracted from NDD CNVs but had no overlap with genes from the CA CNVs at two developmental stages in human tissues (Fetal and Adult) by using high-resolution genome-wide Fourier-transform mass spectrometry data containing in-depth proteomic profiling of 30 histologically normal human samples. Boxplots showing median, interquartile range (IQR) with whiskers adding IQR to the 1st and 3rd quartile, and the line connecting the boxes is comparing the mean expression of the different tissues. Y-axis represents normalised protein expression in spectral counts per gene per sample (SCGS). SCGS, spectral counts per gene per sample (PDF 782 KB) [file 439_2022_2482_MOESM5_ESM.pdf]

# NDD pathogenic deletion genes

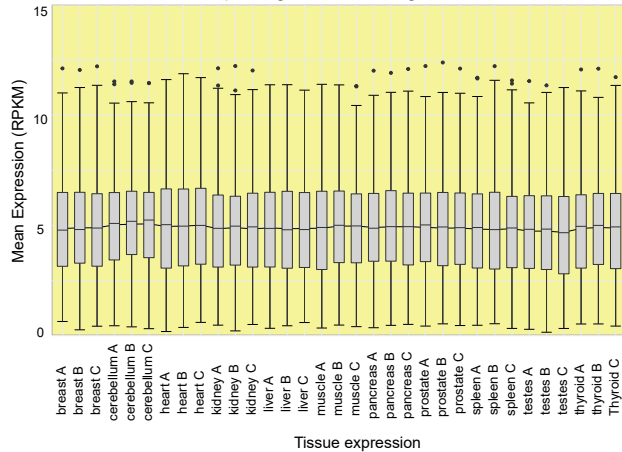

# NDD pathogenic duplication genes

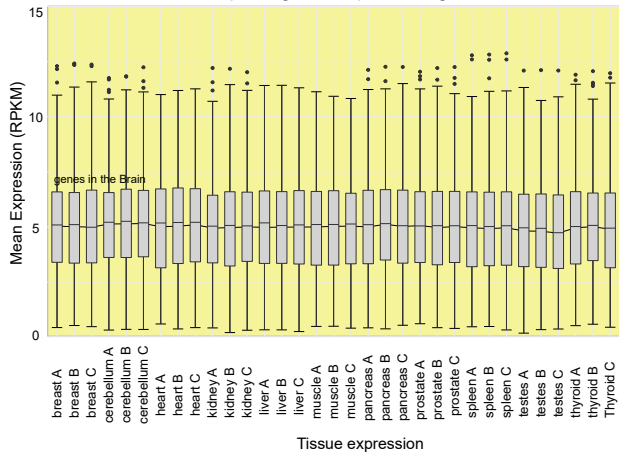

Supplement: Supplementary file 6 — Suppl. Fig. 6: Multi-tissue transcriptome expression analysis of genes from NDD CNVs. Boxplots displaying the expression in multi-tissue transcriptome analysis of genes extracted from NDD CNVs but had no overlap with genes from the CA CNVs. For the multiple tissue expression analysis, we used transcriptomes from 11 normal human tissues (cerebellum, breast, heart, liver, muscle, kidney, thyroid, pancreas, prostate, spleen, and testis) and measured expression levels (in triplicate) displayed in the X-axis. Boxplots showing median, interquartile range (IQR) with whiskers adding IQR to the 1st and 3rd quartile, and the line connecting the boxes is comparing the mean expression of the different tissues. Y-axis represents normalised gene expression in reads per kilobase per million (RPKM) units (PDF 430 KB) [file 439_2022_2482_MOESM6_ESM.pdf]

## Transmembrane ion transport

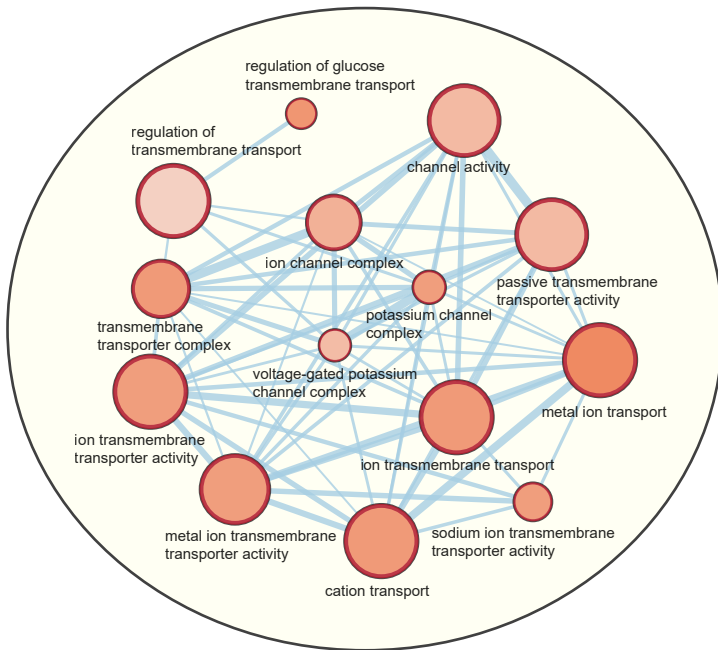

## Photoreceptor cilium activity

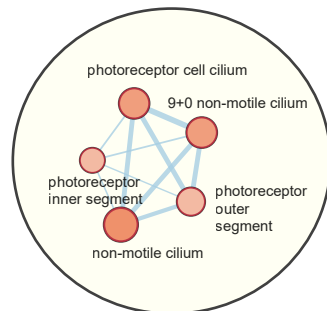

## Organ System development

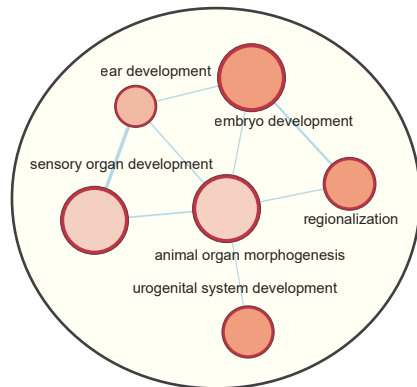

Supplement: Supplementary file 8 — Suppl. Fig. 8: Pathway network map of genes extracted from NDD pathogenic deletion CNVs. Pathway network analysis displaying enriched pathway clusters of genes from NDD pathogenic deletion CNVs that were attained by removing the significant gene overlap with the genes from CA pathogenic deletion CNVs, drawn using Cytoscape. The analysis of significant overlapped loss pathogenic genes with significant pathways (P-value < 0.05) with a false discovery rate (FDR) < 0.01. The color gradient and size of the node represented the P-value and odds ratio, respectively (PDF 527 KB) [file 439_2022_2482_MOESM8_ESM.pdf]

## Synaptic transmission

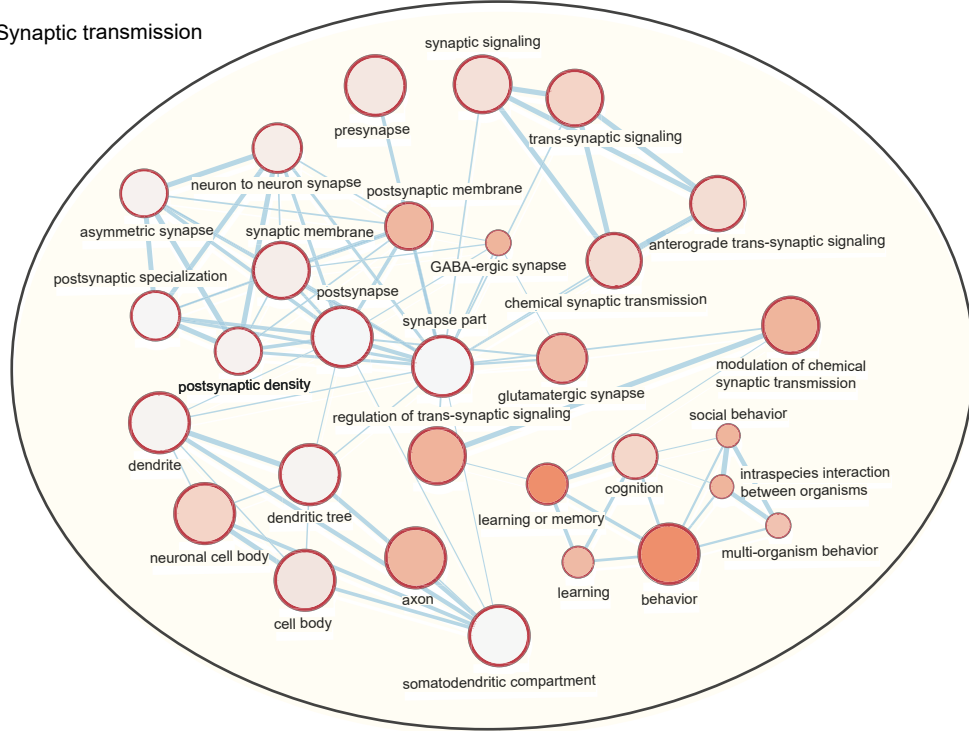

## Morphogenesis and Differentiation

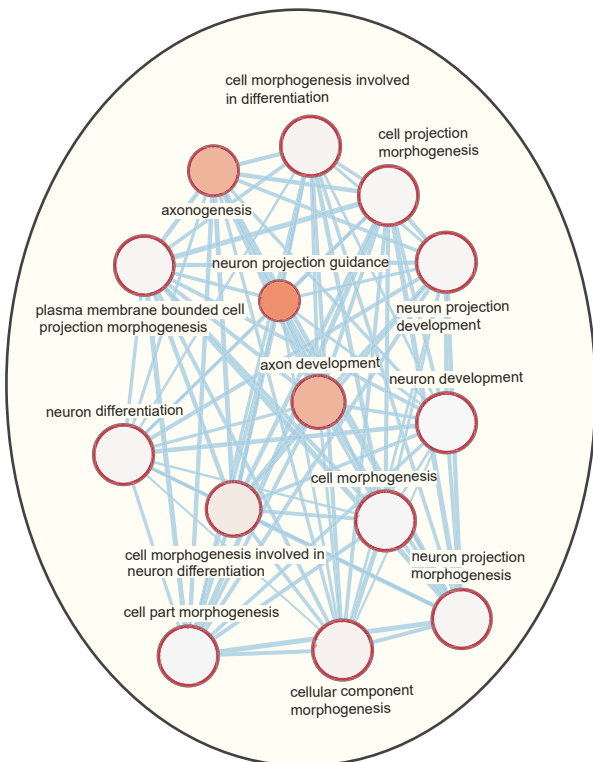

## Catabolic Activity

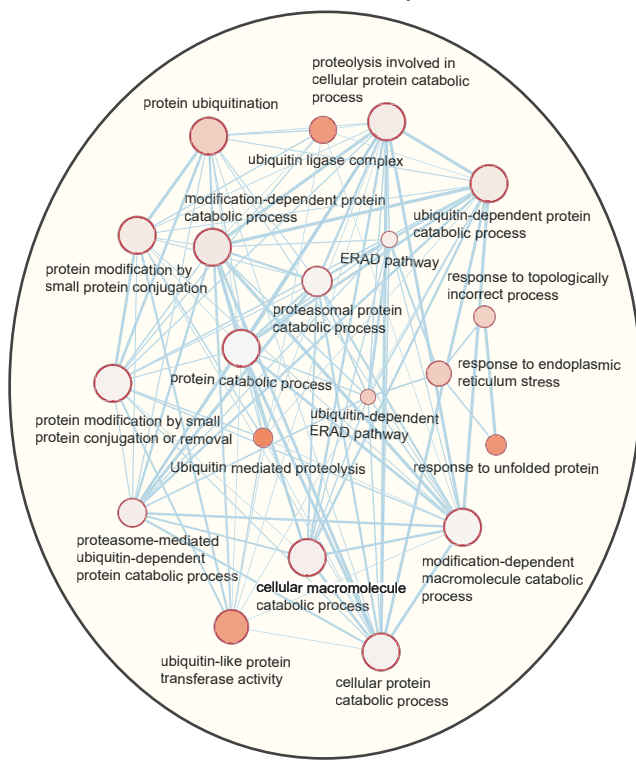

Supplement: Supplementary file 9 — Suppl. Fig. 9: Pathway network map of constraint overlapped genes from pathogenic deletion CNVs. Pathway network analysis in constraint overlapped genes from pathogenic deletion CNVs, drawn using Cytoscape. The analysis of significant overlapped loss pathogenic genes with significant pathways (P-value < 0.05) with a false discovery rate (FDR) < 0.01. The color gradient and size of the node represented the P-value and odds ratio, respectively (PDF 5206 KB) [file 439_2022_2482_MOESM9_ESM.pdf]
