# Supplementary material for: Overlapping pathogenic de novo CNVs in neurodevelopmental disorders and congenital anomalies impacting constraint genes regulating early development
Source: Hum Genet. 2022 Nov 16;142(8):1201–13. doi: 10.1007/s00439-022-02482-5 (PMC10449996; doi:10.1007/s00439-022-02482-5)
Supplement: Supplementary file 4 — Suppl. Fig. 4: Overlapped de novo CNVs phenotypes. Pie chart displaying the count and percentage of characteristic phenotypes of the CNVs that contained constraint overlapped gene lists (filtered with CE OR pLI) between neurodevelopmental disorder (NDD) and congenital anomaly (CA) CNVs. The inner pie chart are CNVs from female cases and the outer pie chart are CNVs from male cases. The three phenotypes seen in descending order of prevalence are developmental delay (Total number: 30 CNVs (60% of overlapped de novo CNVs phenotypes), multiple congenital anomalies (Total number: 19 CNVs (38% of overlapped de novo CNVs phenotypes)), and autism (Total number: 1 CNV (2% of overlapped de novo CNVs phenotypes)) (PDF 373 KB) [file 439_2022_2482_MOESM4_ESM.pdf]

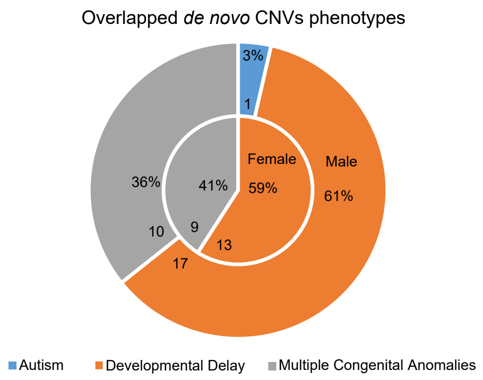
Overlapped *de novo* CNVs phenotypes
3%
1
Female
59%
13
41%
9
Male
61%
17
36%
10
Autism
Developmental Delay
Multiple Congenital Anomalies
