# Supplementary material for: Overlapping pathogenic de novo CNVs in neurodevelopmental disorders and congenital anomalies impacting constraint genes regulating early development
Source: Hum Genet. 2022 Nov 16;142(8):1201–13. doi: 10.1007/s00439-022-02482-5 (PMC10449996; doi:10.1007/s00439-022-02482-5)
Supplement: Supplementary file 7 — Suppl. Fig. 7: Spatiotemporal association analysis of critical exons in constraint genes. Boxplots displaying the spatiotemporal transcriptome data of constraint overlapped genes extracted from pathogenic deletion and duplication CNVs. Boxplots showing median, interquartile range (IQR) with whiskers adding IQR to the 1st and 3rd quartile, and the line connecting the boxes is comparing the mean expression of the different tissues. Y-axis represents normalised gene expression in reads per kilobase per million (RPKM) units (PDF 687 KB) [file 439_2022_2482_MOESM7_ESM.pdf]

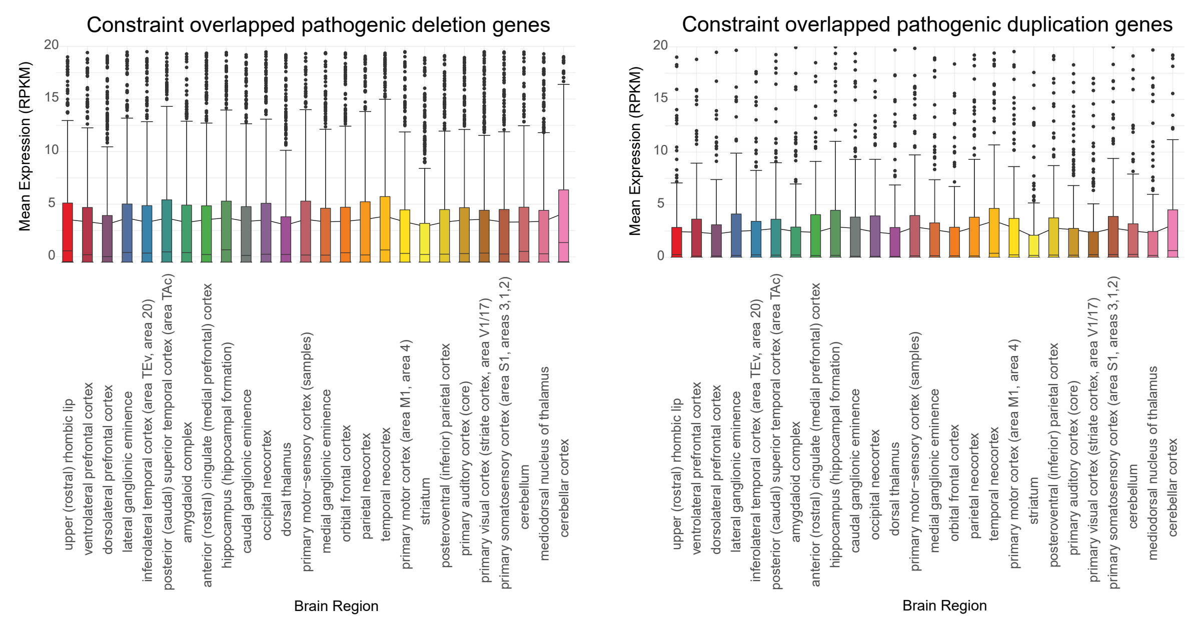

Constraint overlapped pathogenic deletion genes
Mean Expression (RPKM)
20
15
10
5
0
upper (rostral) rhombic lip
ventrolateral prefrontal cortex
dorsolateral prefrontal cortex
lateral ganglionic eminence
inferolateral temporal cortex (area TEv, area 20)
posterior (caudal) superior temporal cortex (area TAc)
amygdaloid complex
anterior (rostral) cingulate (medial prefrontal) cortex
hippocampus (hippocampal formation)
caudal ganglionic eminence
occipital neocortex
dorsal thalamus
primary motor−sensory cortex (samples)
medial ganglionic eminence
orbital frontal cortex
parietal neocortex
temporal neocortex
primary motor cortex (area M1, area 4)
striatum
posteroventral (inferior) parietal cortex
primary auditory cortex (core)
primary visual cortex (striate cortex, area V1/17)
primary somatosensory cortex (area S1, areas 3,1,2)
cerebellum
mediodorsal nucleus of thalamus
cerebellar cortex
Brain Region
Constraint overlapped pathogenic duplication genes
Mean Expression (RPKM)
20
15
10
5
0
upper (rostral) rhombic lip
ventrolateral prefrontal cortex
dorsolateral prefrontal cortex
lateral ganglionic eminence
inferolateral temporal cortex (area TEv, area 20)
posterior (caudal) superior temporal cortex (area TAc)
amygdaloid complex
anterior (rostral) cingulate (medial prefrontal) cortex
hippocampus (hippocampal formation)
caudal ganglionic eminence
occipital neocortex
dorsal thalamus
primary motor−sensory cortex (samples)
medial ganglionic eminence
orbital frontal cortex
parietal neocortex
temporal neocortex
primary motor cortex (area M1, area 4)
striatum
posteroventral (inferior) parietal cortex
primary auditory cortex (core)
primary visual cortex (striate cortex, area V1/17)
primary somatosensory cortex (area S1, areas 3,1,2)
cerebellum
mediodorsal nucleus of thalamus
cerebellar cortex
Brain Region
